# Supplementary material for: Unlocking the mentally ill in Indonesia: An empirical study of the effectiveness of a “Bebas Pasung” program in Central Java
Source: PLoS One. 2024 Oct 18;19(10):e0302190. doi: 10.1371/journal.pone.0302190 (PMC11488725; doi:10.1371/journal.pone.0302190)
Supplement: Attachment — Submitted filename: Response to Reviewers.docx [file pone.0302190.s001.docx]

July 6^th^, 2024

Dear Dr. Khobragade,

We deeply appreciate all the effort you and your reviewers have made in reviewing this study and making suggestions to clarify and strengthen the manuscript. We have made every effort to respond to all suggestions and use them to improve the manuscript.

Journal requirement:

1. We have attempted follow all style requirements, including file naming.
2. We have revised the Funding Statement and include it in the cover letter as suggested. Here is the full version of Funding Statement:

This research is supported by USAID Cooperative Agreement No. AID-497-A-11-00017, "Inter-University Partnerships for Strengthening Health Systems in Indonesia: Building New Capacity for Mental Health Care" (https://www.usaid.gov/id/indonesia ). This was a USAID Cooperative Agreement with Harvard Medical School, with BJG as Project Director and MJDG as Co-Project Director. Subcontracts to Gadjah Mada University, with MAS and CRM as Principal Investigators, supported research activities in Yogyakarta from 2011 through 2014. Data analysis and writing was partially supported by a grant to the Department of Global Health and Social Medicine from the Harvard Medical School Center for Global Health Delivery--Dubai, for a project entitled "Building a Model for Comprehensive Mental Health Care in Yogyakarta, Indonesia." Harvard Principal Investigators were BJG and MJDG. Work in Indonesia was supported by a consortium agreement with Gadjah Mada University, with MAS and CRM as Principal Investigators. The funders had no role in study design, data collection and analysis, decision to publish, or preparation of the manuscript. There was no additional external funding received for this study.

3. We have made arranged for the Research Ethics Committee of the Faculty of Psychology, Gadjah Mada University, to be responsible for making the data available according to current norms.

Here is the full Data Availability Statement:

For approved reasons, some access restrictions apply to the data underlying the findings. Given the sensitivity of the data and the fact that data are not formally de-identified owing to on-going research, sharing of the data requires IRB approval for researcher who meet the criteria for access to confidential data. Data on the specific variables in this study are available upon request and requests may be sent to the Head of Research Ethics Committee, Faculty of Psychology Universitas Gadjah Mada, email: [komisietikpsi.psikologi@ugm.ac.id](mailto:komisietikpsi.psikologi@ugm.ac.id)

4. Interview transcripts and detailed hospital record data cannot be made available due to sensitive information that may jeopardize the identity of participants. However, data on the specific variables in this study are available upon request.
5. We have reviewed the reference list for completeness and format. None of the cited papers have been retracted, to our knowledge.

Additional Editor Comments

We appreciate the overall positive tenor of the comments, and we have responded to the two specific suggestions.

1. We have placed all of the data describing the sample from the first eight tables into the text. All remaining eight tables are cited in the text.
2. We have added information about the statistical analyses in the Methods Section (in lines 157-162).

Review comments by Reviewer 1 and 2

Basic comments (1-4) by both reviewers.

We very much appreciate the overall positive tenor of this review.

1. *Is the manuscript provided technically sound and does the data support the conclusions?*

Response: no additional revisions are suggested.

2. *Has the statistical analysis been performed appropriately and rigorously?*

*Comments: However, the manuscript could benefit from more detailed information on the statistical methods used for analysis, such as specific test performed*.

Response: As described above, added information concerning statistical methods used for analysis has been added in lines 157-162.

3. *Have the authors made all data underlying the findings in their manuscript fully available?* Response: As described in response to the Journal Requirements item 3, we have now made arrangements for the de-identified data to be made available in a fashion that meets the PLOS Data policy that the Research Ethics Committee of the Faculty of Psychology of Gadjah Mada University will be the responsible sight for the data. The hospital records cannot be made available, given confidentiality of clinical data, and the interview transcripts cannot be adequately de-identified. However, all data coded and drawn from these sources will be available on request to the Research Ethics Committee office.
4. *Is the manuscript presented in an intelligible fashion and written in standard English?*

*Comments: Minor editorial revisions could further refine the manuscript’s clarify and presentation quality.*

Response: The manuscript had undergone additional editorial revision for clarity and presentation quality by the primary American PIs on the original study, both of whom have been journal editors.

Summary and Recommendations

**Reviewer #1**

Again, we deeply appreciate the fact that this reviewer finds the results and analyses to be of importance for understanding the care of the mentally ill in low resource settings and for the development of further research aimed at improving policies and services.

Suggestions:

*Provide more detailed descriptions of the statistical methods.*

We have responded to this above; added information provided in lines 157-162.

*Clarify data availability*

See above.

*Undergo minor editorial revisions*

See above.

**Reviewer #2**

We deeply appreciate this reviewer's overall comments about the importance of this study and what this reviewer learned personally from this manuscript. We agree, of course, that although very different, Western societies face equally tragic lack of genuine care for persons with severe mental illness. In the United States, we recognize that "pasung" or confinement often means the carceral system or homeless shelters, and that "return to the community" often has extraordinarily tragic outcomes.

1. *Could you provide a brief explanation of the Bebas Pasung policy, focusing on its legal, administrative and organization aspect in the introduction*?

Response: We have added information concerning the Bebas Pasung policy both at the national level and in the Province of Central Java in lines 98-99 and 119-123. Indonesia’s Bebas Pasung initiative was launched in 2010 by the division of Mental Health of the Ministry of Health of Indonesia. This initiative was not followed by a formal policy or regulations that were binding. There is no obligation for provincial or district governments to respond. As noted, the 2010 aspirational policy declaration was followed by the passage of Indonesia's National Mental Health Law in 2014. However, that law was never implemented or turned into regulations, as required by the law, and has now been replaced by what is known as the Omnibus Bill. Fortunately, the provincial government of Central Java responded by the development of a provincial initiative, that led to the Bebas Pasung Program described in this study.

2. *Could you elaborate on how the data might be interpreted within the broader context of potential future interventions aimed at enhancing compliance and preventing re-locking?*

Response: Full response to this issue would and perhaps will require a full paper, further intervention trials and research, and many years of continued work. We have discussed the relevance of this study for overall mental health services policy and design, adding further detail, in line 475-480, 486-491, and 501-510 of the Discussion and lines 530-533 of the Conclusion.

Potential future interventions aimed at enhancing treatment compliance and reducing relocking as much as possible include strengthening the community-based mental health services that promote community-based rehabilitation programs, with a focus on further training of Community Mental Health Nurses in the primary care system and added training and funding of village mental health cadre.

3. *Based on your data, what types of support or interventions do you suggest could be implemented to improve both the family’s circumstances and patients’ outcomes*?

Response: Again, as noted in response to comment 2, we have attempted to address these issues in broad terms. A longer response would include the importance and limitations of the National Health Insurance system (e.g. the fact that its implementation allows mental hospital to limit hospital stays to as little as 7-10 days), the need for recovery-oriented training of physicians and nurses in the national primary health system, and also financial support for families caring for members with severe mental illness. Obviously, many of these issues are beyond the scope of this research report. We appreciate this reviewer's concern for these issues. However, we have tried to focus on the specific findings of this study and the immediate implications for mental health policy.

4. *Is it possible to compare your findings with data from a “typical” psychiatric population within your country to high light any unique trends or differences*?

Response: It is somewhat unclear what this suggestion means. We believe the psychiatric population of Central Java is not atypical, although Indonesia is vastly varied in local cultural differences and in access to services. Far more studies are needed to give a more comprehensive view of conditions around Indonesia. We have gestured to this issue both by noting the recent literature review of *pasung* in Indonesia and by noting the importance of far more research in this domain.

We also feel that the explicit comparison of the data from this study with data from a much longer study in China, in which the overall PIs of this program of research were also involved, adds significantly to our ability to speak to both the strengths and weaknesses of this program in Indonesia.

5. *Are there more socio-economic data available about the families involved in the study that could be shared?*

Response: We responded to this by adding information in lines 304-307.

We asked the family caregivers how they defined their economic condition. 50% said they were poor or very poor, the remaining half said they were average.

6. *Do you have additional clinical data about bout the studied population that could shared? (e.g. CGI-S scores at program entry and CGI-I scores at exit?)*

Response: Unfortunately, we were not able to make any further evaluation of current psychiatric symptom levels of patients at the time of the home interviews. This is of course an additional limitation of the study. We added a note about this in lines 514-518.

7. *Additionally, would it be possible to anonymize the data using a feasible method to facilitate sharing with fellow researchers*?

Response: Yes. We have made arrangements to have data made available, as described above.

8. *To enhance the methodological rigor of your results, I suggest incorporating a brief statistical methods paragraph that outlines the statistical significance test you’ve conducted.*

Response: We have done so as noted above.

Overall, we have made every effort to address all issues raised by the reviewers, as well as the editorial staff. We appreciate enormously their suggestions, and we hope our revisions have strengthened the manuscript.

With best wishes

Tri Hayuning Tyas (Corresponding author)
